# MRI perfusion analysis using freeware, standard imaging software

**DOI:** 10.1186/s12917-020-02352-0

**Published:** 2020-05-18

**Authors:** Antje Hartmann, Volkher B. Scholz, Ines E. Lautenschläger

**Affiliations:** 1 Veterinary Clinic Hofheim, Katharina-Kemmler-Str. 7, 65719 Hofheim, Germany; 2 Department of Physics and Astronomy, Ghent University, Krijgslaan 281, Ghent, Belgium; 3 Vetsuisse Faculty, University of Zürich, Clinic for Diagnostic Imaging, Winterthurerstrasse 260, 8057 Zürich, Switzerland

**Keywords:** Magnetic resonance imaging, Canine, Brain, PWI, OsiriX®, Horos®, CBF, Machine learning, R package

## Abstract

**Background:**

Perfusion-weighted imaging is only scarcely used in veterinary medicine. The exact reasons are unclear. One reason might be the typically high costs of the software packages for image analysis. In addition, a great variability concerning available programs makes it hard to compare results between different studies. Moreover, these algorithms are tuned for their usage in human medicine and often difficult to adapt to veterinary studies.

In order to address these issues, our aim is to deliver a free open source package for calculating quantitative perfusion parameters. We develop an “R package” calculating mean transit time, cerebral blood flow and cerebral blood volume from data obtained with freely imaging software (OsiriX Light®). We hope that the free availability, in combination with the fact that the underlying algorithm is open and adaptable, makes it easier for scientists in veterinary medicine to use, compare and adapt perfusion-weighted imaging analysis.

In order to demonstrate the usage of our software package, we reviewed previously acquired perfusion-weighted images from a group of eight purpose-breed healthy beagle dogs and twelve client-owned dogs with idiopathic epilepsy. In order to obtain the data needed for our algorithm, the following steps were performed: First, regions of interest (ROI) were drawn around different, previously reported, brain regions and the middle cerebral artery. Second, a ROI enhancement curve was generated for each ROI using a freely available PlugIn. Third, the signal intensity curves were exported as a comma-separated-value file. These files constitute the input to our software package, which then calculates the PWI parameters.

**Results:**

We used our software package to re-assess perfusion weighted images from two previous studies. The clinical results were similar, showing a significant increase in the mean transit time and a significant decrease in cerebral blood flow for diseased dogs.

**Conclusion:**

We provide an “R package” for computing the main perfusion parameters from measurements taken with standard imaging software and describe in detail how to obtain these measurements. We hope that our contribution enables users in veterinary medicine to easily obtain perfusion parameters using standard Open Source software in a standard, adaptable and comparable way.

## Background

In a medical context perfusion is defined as the blood volume that passes through a capillary bed in a defined time period [1, 2]. Perfusion-weighted imaging (PWI) is a set of techniques to visualize the blood volume flow for medical examinations. It requires a tracer that visualizes the blood flow dynamics and can be imaged over time. This tracer can be endogenous or exogenous of origin. Arterial spin labeling (ASL) is performed using blood as endogenous tracer: the blood is labeled by an inversion or saturation pulse, leading to a change in signal intensity if it enters the slice(s) of interest [1]. In comparison, a more robust way of performing perfusion measurements is employing an exogenous contrast medium as tracer. The most commonly used contrast medium is a gadolinium chelate, which is injected intravenously as a bolus [1].

The acquisition of perfusion images is then performed using one of two basic techniques. In dynamic-contrast enhanced magnetic resonance imaging (DCE -MRI) the increase in signal intensity due to T1-effects is extracted from T1-weighted sequences. This technique is particularly suited for assessing the contrast medium kinetics within a tissue over a longer time period, and is hence mainly used in tumor assessment [1, 3, 4]. The second technique, called dynamic-susceptibility-contrast magnetic resonance imaging (DSCE-MRI) uses dynamic T2*-weighted sequences to evaluate regional, cerebral perfusion parameters by detecting signal loss due to T2* effects caused by the contrast medium [1]. For this, dynamic images are acquired before, during, and after contrast medium injection [1, 5, 6].

Both techniques are followed by the extraction of signal intensity–time curves from the acquired images. In a next step, these are converted into concentration-versus-time curves, from which several quantitative perfusion parameters can be obtained. Typical examples are the time of arrival (T0), the time to peak (TTP), the mean transit time (MTT), the cerebral blood flow (CBF) and the cerebral blood volume (CBV) [1], which are defined as follows: T0 and TTP are time parameters representing the time between the injection of the contrast medium and its arrival at the region of interest (ROI) (T0) and the time to its maximum concentration (TTP), respectively. The MTT describes the average amount of time the contrast agent takes to pass through the ROI [1, 7]. The CBF is defined as the blood flow through a region-of-interest (ROI) divided by the mass of that ROI [2, 5, 7]. The CBV is the product of MTT and CBF and hence gives the volume of blood in a ROI divided by the mass of the ROI [5]. After their calculation, these parameters can be used to generate colored perfusion maps allowing for qualitative assessment of brain perfusion, e.g. by comparing the right and left hemisphere.

On the technical side, the extraction of certain of these perfusion parameters, namely MTT, CBF and CBV, from the magnetic resonance (MR) images is based on a mathematical technique called deconvolution [8]. Several mathematical techniques are available for performing this step, see the review by Fieselmann et al. [8]. However, the exact implementation of these algorithms is vendor specific and often not available for review, resulting in the fact that the obtained parameters cannot be easily compared between different MR systems [9, 10]. Moreover, the software packages performing these calculations are usually very expensive and often specifically designed for use in human medicine. These restrictions motivated us to provide an open source implementation of a deconvolution algorithm for use in veterinary perfusion imaging.

### Implementation

We implemented our algorithm in R^1^. Being commonly used in statistical computing, R also supports numerous numerical and statistical tasks. We chose it compared to other software packages (e.g. Matlab) because it is distributed as open-source software and thus freely available. Consequently, we provide our software as an “R package”, available online on the Open Source platform “GitHub”, https://github.com/volkherscholz/vetperf. More detailed information on how to use this package is available online, as well as through the usual “R” help functionality. Our deconvolution algorithm is based on the ideas reviewed in the contribution of Fieselmann et al. [8] and focuses on extracting the MTT, the CBF as well as the CBV from data obtained from employing an exogenous contrast media such as gadolinium as tracer. An extension to compute also TTP and T0 is straightforward and planned for the next release. In order to describe the main idea of the algorithm, we briefly review the physical mechanism.

The calculation of perfusion analysis is based on measurements taken while the bolus travels through the blood system. The starting point is the arterial input function (AIF), measured at the artery before the contrast agent bolus travels through the region of interest (ROI). It is inversely proportional to the density of the contrast agent bolus in the blood. The received signal in the ROI is a combination of the AIF with the internal ROI structure. The mathematical operation modelling this relationship is called convolution. More specifically, the output signal is obtained from convolving the arterial input function with the flow-scaled residue function (see reference [8] for details). In order to extract the perfusion parameters, which depend on the internal ROI structure (encoded in the flow-scaled residue function), we have to reverse this mathematical operation, a process called deconvolution.

**Fig. 1:**
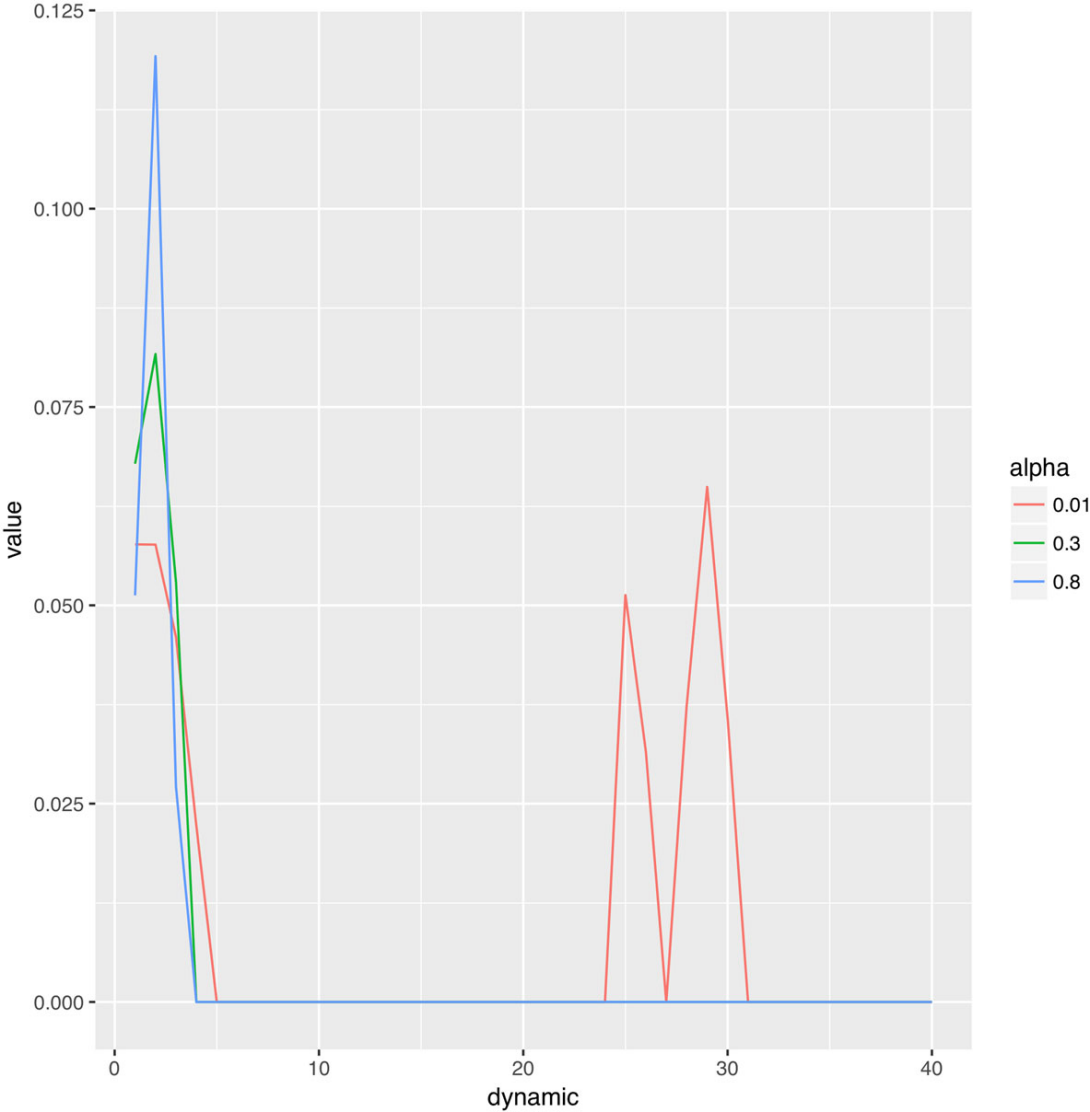
Visualizations of computed flow-scaled residue functions for different values of the hyperparameter alpha. We see that for very low alpha (alpha = 0.01) the computed result shows unphysical spikes at later dynamical steps. Even for reasonable small alpha (alpha = 0.3) the computed flow-scaled residue function does not show these spikes. This effect is even more pronounced for high alpha (alpha = 0.8) x-axis (dynamic): number perfusion-weighted dynamic image obtained per slice. For each slice 40 dynamics were acquired, being acquired 1.6 s apart between each other. Dynamic image 1–9 provide the baseline information, dynamic image 10–40 show contrast medium flow dynamics. y-axis (value): value of the computed flow-scaled residue function.

The review article [8] explains several of mathematical techniques for performing this operation, focusing on an algorithm based on L2 weighted regression (Ridge regression). We implemented an extension of this algorithm, using ideas from Machine Learning. More specifically, our package employs the elastic-net algorithm [11] from the “R package” “glmnet” [12, 13] to perform the deconvolution. Compared to just ridge regression, elastic-net enforces that the obtained flow-scaled residue function is “sparse”, i.e. does not have too many non-zero entries. In addition, we can enforce that it should only have non-negative values. Together, both constraints typically lead to flow-scaled residue functions with less high order fluctuations than those obtained from just ridge regression (see Fig. 1). The elastic-net algorithm features two hyperparameters, usually referred to as alpha and lambda, which control the exact terms in the objective function (see [11, 12] for details). The hyperparameter alpha ranges from 0 to 1 and concerns the combination of ridge regression and sparsity penalties - a small alpha puts more emphasis on ridge regression, while a larger alpha enforces sparsity stronger. The hyperparameter lambda scales the entire regression terms, involving both the ridge as well as the sparsity constraint. We follow the standard approach (see [12, 13]) and let the user specify the alpha parameter while automatically choosing lambda. More specifically, we perform cross-validation: we break the data apart into random subsets, then fitting the data several times for different values of lambda, with different subsets omitted. The package glmnet then chooses the best lambda out of these runs. In order to reduce the variability due to the random nature, we repeat that process several times (default are 10 iterations) and use the resulting average value for lambda. The variability can be further reduced by performing more iterations, with the consequence that the algorithm is slower.

Apart from the basic deconvolution algorithm, our package provides convenience functions which allow the user to input the data in terms of tables obtained from reading in comma-separated-values (CSV) files. The software package can also be employed to compute perfusion parameters normalized by a ROI of choice (i.e. with measurements obtained in the white matter). We used our algorithm to re-assess data obtained from dogs from previous studies. The data is included in the “R package” and its analysis is also documented in a package vignette. We now briefly review its content.

### Data set

The dataset consists of measurements acquired from images from eight healthy and twelve dogs with idiopathic epilepsy obtained within two previous research projects were assessed [14, 15]. MRI was performed using a 1.0 Tesla^2^ superconductive system and a SENSitivity Encoding (SENSE) coil^3^. The following sequences were acquired in addition to the PWI: dorsal and transverse T2-weighted images, transverse T2 weighted FLAIR images, transverse T2*-weighted gradient echo images, transverse T1-weighted sequence pre- and post-contrast medium administration and dorsal T1-weighted gradient echo images pre- and post-contrast medium administration. We performed DSC-MRI employing a dynamic multishot fast-field-echo echo-planar sequence in a dorsal plane, paralleling the skull base with one slice through the thickest portion of the caudate nucleus. For each slice 40 dynamics were acquired, being acquired 1.6 s apart between each other [14, 15]. We used gadoteric acid as a contrast agent, which was injected intravenously at the 10th dynamic at a dose of 0.2 mmol/kg body weight. The injection was performed using a double-headed injection pump with an injection rate of 5 ml/second, followed by an injection of 20 ml of Ringer’s solution. Thus, the first nine images provided the baseline information, while the images from dynamic time step ten and onwards showed contrast medium flow dynamics [14, 15].

### Image and data processing

**Fig. 2:**
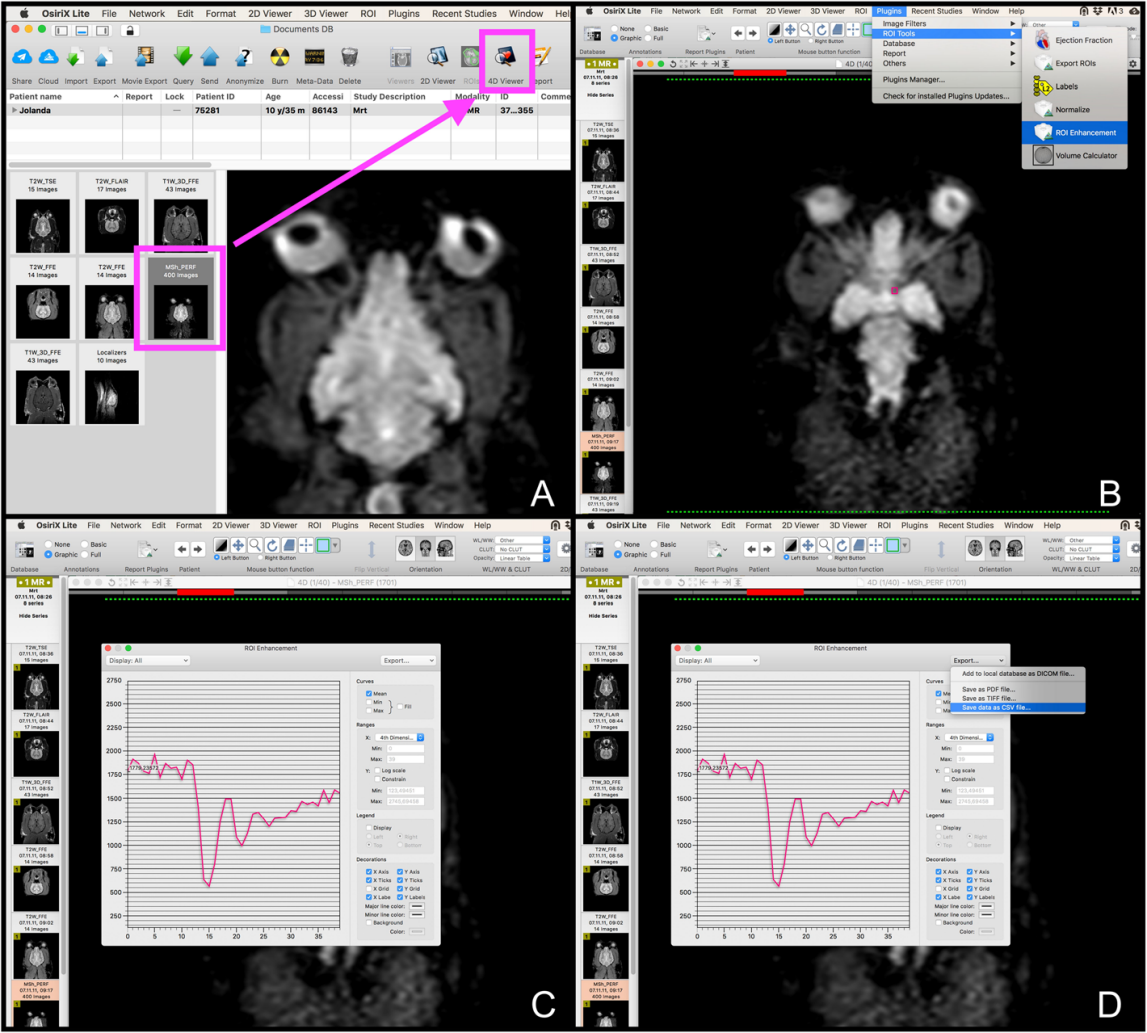
Step-by-step description of how signal intensities can be extracted from perfusion-weighted images using OsiriX Light®. First, the respective sequences must be opended using the 4D-Viewer (**a**). Second, a ROI is drawn around the anatomy of interest (small pink rectangle visible on (**b**)). Third, one choses from the PlugIn drop-down menu the item ‘ROI enhancement’ (**b**). Forth, a signal intensity-curve is generated for the ROI (**c**). The individual signal intensities can then be exported as a CSV file

**Fig. 3:**
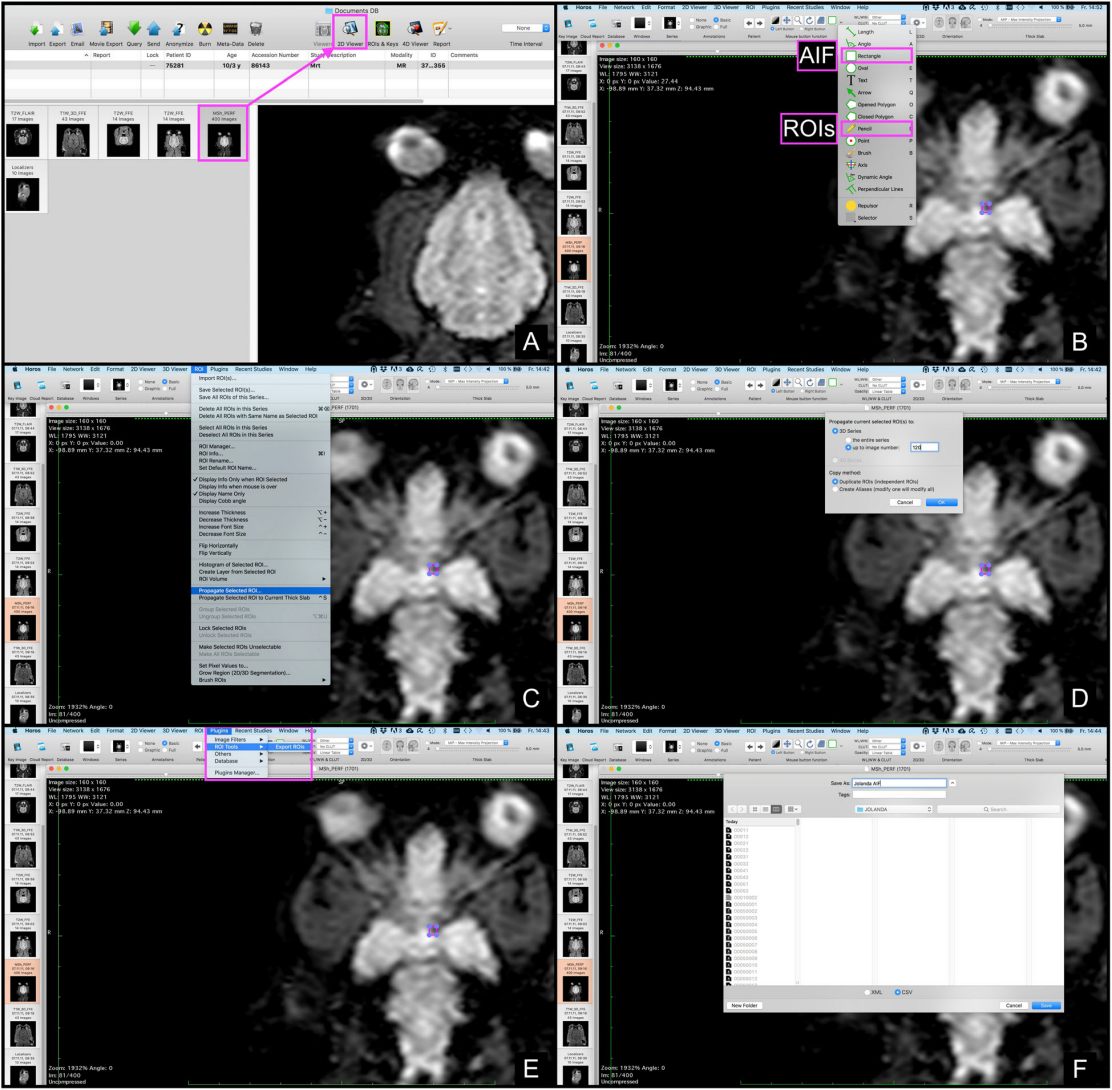
Step-by-step description how signal intensities can be extracted from perfusion-weighted images using Horos®. Horos does not provide a ‘ROI enhancement’ PlugIn, but it is nevertheless possible to obtain dynamic perfusion data. First, the respective sequences must be opend using the 2D-Viewer (**a**). Second, a ROI is drawn around the anatomy of interest (small pink rectangle visible on (**b**)). Importantly, the ROI must be drawn on the first dynamic image showing the anatomic region of interest. We decided for a rectangular ROI for the AIF and the pencil tool for the anatomic regions (**b**). Third, one chooses the option ‘propagate selected ROI’ from the drop down menu ‘ROI’ (**c**). The ROI must be propagated up to the last dynamic image of the respective slice (therefore you need to start at the first slice). In our case, we obtained 40 dynamic images per slice, thus starting at slice 81 we need to propagate the ROI up to slice number 120 (**d**). Fourth the options ‘ROI tools’ and ‘Export ROI’ need to be selected from the ‘PlugIn’ drop down menu (**e**). This allows for an export of the data as a CSV file (**f**)

In order to compute the perfusion parameters with our “R package”, the images obtained within the previous studies were imported into the viewing software (OsiriX Light®^4^) and opened using the 4D-Viewer. In the 4D-Viewer, the navigation through the different slices is possible using the right and left arrow keys, while the selection of the dynamic steps is performed employing the up and down arrow keys. In a next step, ROIs were manually drawn around the caudate nucleus, the thalamus, the piriform lobe including the amygdala, as well as the hippocampus, the semioval center and also the temporal cerebral cortex, using the pencil tool to outline the respective ROI by the first author of this study. Each structure was marked on the left and right and hand side. In analogy to the previous studies, each ROI was outlined on one representative slice, and chosen to be as large as possible, while avoiding inclusion of adjacent structures [14, 15]. The images of the other sequences were available at the time of the ROI placement and served as a reference for anatomic comparison. The middle cerebral artery was chosen for the AIF, using a rectangular ROI. In the final image processing step, the PlugIn ‘ROI-enhancement’, which is freely available using this viewing software, was used to generate a time-intensity curves. The specific settings were as follows: Curves – mean, X – 4th Dimensions and Y – no decision made. Finally, the intensity data was exported as comma-separated-value (CVS) file, which is a type of spreadsheet file. The Fig. 2a – d visualize the individual steps starting from the ROI selection and ending with the data export. The Fig. 3a – f illustrate the steps necessary to extract the dynamic perfusion data using Horos®^5^, an open source imaging software commonly used in veterinary medicine, which does not offer a ROI-enhancement tool. The collected data is available in the supplementary material (additional file 1) as well as provided with the package itself. In order to compute the MTT, the CBF and CBV, several additional parameters are needed, which are summarized below in Table 1.

**Table 1:** Sequence parameters required by our software package

Parameter	Value
time of echo (TE)	30 ms
time bin	1.6 ms
arrival frame	14.4 ms

**Fig. 4:**
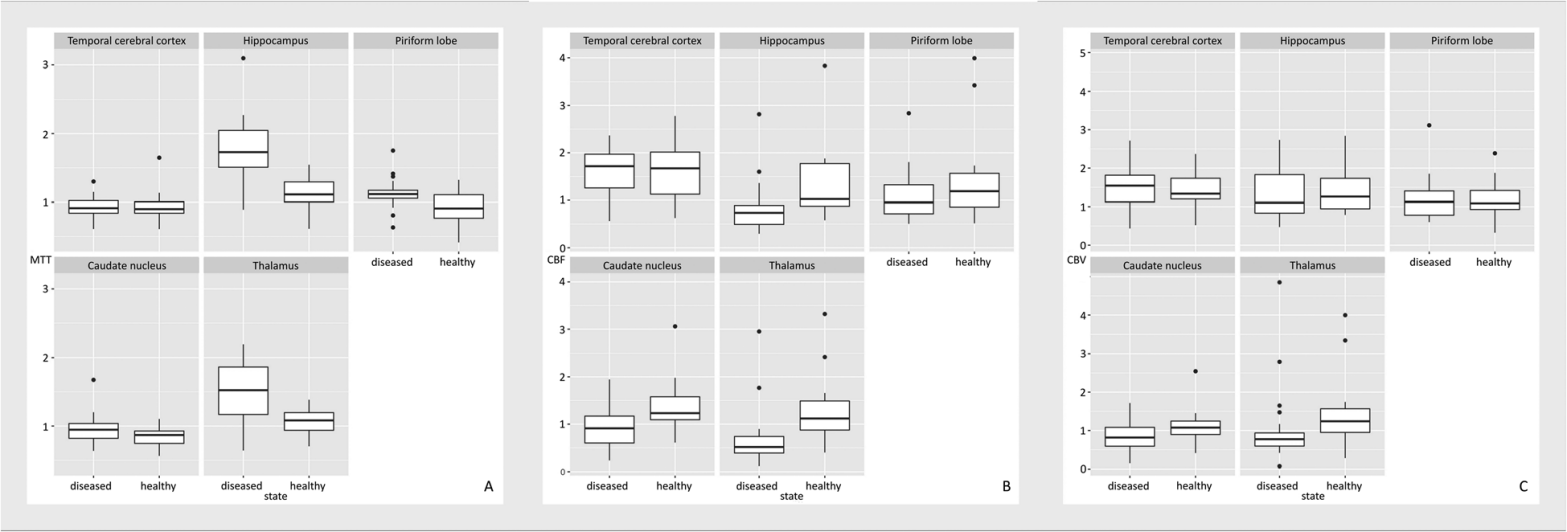
Boxplots comparing the mean transit time (MTT -- **a**), the cerebral blood flow (CBF -- **b**) and the cerebral blood volume (CBV -- **c**) for five different regions of interests (ROIs) between diseased and healthy dogs. All values are normalized with respect to the Centrum semiovale and were computed using a choice of alpha = 0.3. We clearly see a tendency of a higher mean transit time and a lower cerebral blood flow for diseased dogs, most prominently for the Hippocampus and the Thalamus. The box represents the interquartile range of the respective value, with the median value been shown as a vertical line within the box. The whiskers show the lower and upper extreme. The single data points represent the outliners. For example, in Fig. 4a, the box shows the interquartile range for the MTT for different regions named in the top row of each individual graph for diseased and healthy dogs.

**Fig. 5:**
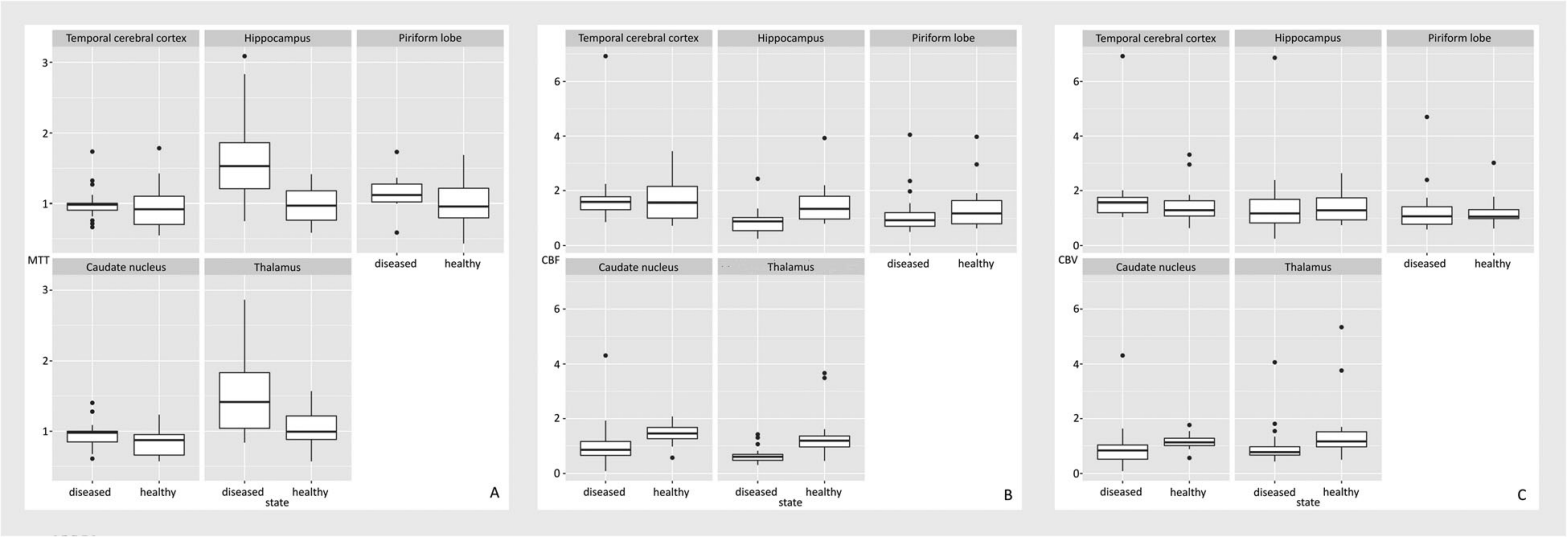
Boxplots comparing the mean transit time (MTT -- **a**), the cerebral blood flow (CBF -- **b**) and the cerebral blood volume (CBV -- **c**) for five different regions of interests (ROIs) between diseased and healthy dogs. All values are normalized with respect to the Centrum semiovale and were computed using a choice of alpha = 0.8. We clearly see a tendency of a higher mean transit time and a lower cerebral blood flow for diseased dogs, most prominently for the Hippocampus and the Thalamus. Overall the boxplots show a very similar behavior as for the value alpha = 0.3. The properties of the box plots are identical to Fig. 4.

We used our package to compute relative perfusion parameters (normalized by the measurement taken from the semioval center) for all 20 dogs, both sides and all five ROIs.

(ROIs (see Fig. 4a - c). Our choice for the hyperparameter alpha was 0.3, expressing a tendency towards ridge regression while still enforcing sparsity. However, we did observe that different choices of alpha did not influence the clinical results (see Fig. 5a - c for the choice alpha = 0.8).

In order to assess whether the parameters returned by our algorithm are still clinically useful, we checked whether a significant difference can be seen difference between healthy and diseased dogs, as observed previously [14, 15].

The comparison was made by fitting (general) Linear Mixed Models (LMM) to the computed parameters MTT, CBF and CBV. All models included a fixed effect modelling the influence of the ROI and a random effect modelling the influence of the animal (the normal distribution was chosen for MTT random effect, while a Gamma variate for chosen for the CBF and CBV, see [15] for a detailed explanation). Half of the models (one each for MTT, CBF and CBV) in addition included a fixed effect expressing the influence of the state (healthy vs diseased). We then compared the fit quality of both models (with and without the effect depending on the state) using the chi-squared test. The results are summarized in Table 2.

**Table 2:** Statistical comparison of healthy and diseased dogs using LMMs. We used the R package lme4 for determining and comparing the models [13]

Parameter	DF	chi-sq	*P*-value
MTT	5	46.138	8.513e-09 ***
CBF	5	22.243	0.0004707 ***
CBV	5	8.501	0.1307

## Results

The results are in good agreement with results obtained previously using perfusion parameters obtained from proprietary software [15]. Absolute values generated using our software package varied from previously generated values using vendor specific software, with a significant increase in the mean transit time and a significant decrease in the cerebral blood flow for diseased dogs. The relationship between the values for healthy and diseased dogs were similar to the previously generated values using vendor specific software.

## Discussion

Perfusion-weighted imaging is currently only scarcely used in veterinary medicine. Image analysis requires costly software packages. Great variability in available programs using different deconvolution algorithms, with non-transparent mathematics behind them, exists. There is no standard recommendation for perfusion software and no quality assurance program testing reliability and accuracy of calculated values is performed. Depending on the disease process varying software are better suited to analyze perfusion data; not every software can deal with every type of perfusion image and although values should theoretically be comparable between different software in reality this is questioned. Thus, doubts concerning the reliability of quantitative perfusion parameters even exist in human medicine [8, 9]. This might lead to further wariness in veterinary medicine preventing investment into perfusion software.

We herein provide an “R package” computing the main perfusion parameters (MTT, CBF and CBV) from measurements taken with standard imaging software and describe in detail how to obtain these measurements.

An in-depth examination and comparison of the parameters obtained from our implementation and those of different MR vendors was out of scope of this study, as it would require licenses of different software packages. As explained above (see also [9, 10]), it is also unclear whether such an examination would return valuable results.

Instead, we chose to repeat the statistical analysis from the previous studies, as reported above. The clinical results obtained with our algorithm are similar to the ones obtained with proprietary software packages [14, 15]. More precisely, we see a significant increase in MTT, and a significant decrease in CBF for diseased dogs as compared to healthy ones, as previously reported. The increased MTT implies a slower blood flow through an individual ROI and corresponds well with the decreased CBF, thus an overall reduced blood flow through a specific ROI. In accordance with our results hypoperfusion, as expressed by the decrease in CBF, characterizes the ictal focus in human patients with temporal lobe epilepsy in the interictal time [16–21]. However, alterations in PWI vary with the cause of epilepsy in human medicine. In hippocampal sclerosis a decreased perfusion interictally has been described [16, 22], which is considered to be the result of cell loss and sclerosis [23]. In veterinary medicine hypometabolism of the cerebral cortex has been shown in a group of epileptic Logotto Romagnole dogs compared to a healthy control group of the same breed FDG-PET [24]. One can speculated that a reduced metabolism is related to the reduced blood flow, however it remains unknown which is sequel of the other.

The lack of a significant difference in CBV is most likely due to the fact that it can be expressed as the product of the two: as the MTT is statistically higher, while the CBF is statistically lower, their product includes two competing effects which hence can lead to less statistically significant differences.

We concede that one statistical analysis does not prove that our implementation shows a comparable performance when put side by side with vendor specific solutions. However, is nevertheless shows the potential of our software package. And being open source, we do encourage the reader to try it out on other datasets and report any potential improvement back to the community and us.

## Conclusion

Functional magnetic resonance imaging of the central nervous is still in its infancy in veterinary medicine. However, it is reasonable to expect that it will gain increasing importance in the near future, given the fact that invasive treatment options require information about brain function in order to exactly address the diseased brain area and to avoid damage to a vital brain area. Furthermore, the increasing availability of refurbished high-field MR system from human medicine makes these techniques affordable to veterinarians.

In order to facilitate the use of perfusion weighted imaging in veterinary medicine, we provide an open source software package for calculating several key quantitative perfusion parameters. As described above, the necessary measurements can be obtained with standard imaging software. Moreover, the computational steps are open source, allowing for an easy comparison and possible adjustment to other situations. We thus hope that our publication leads to an increase usage of PWI in veterinary medicine.

## Availability and requirements

**Project name:** R package for performing perfusion analysis for veterinary studies

**Project home page:** https://github.com/volkherscholz/vetperf

**Operating system(s):** Linux, Windows, Mac OS X

**Programming language:** R

**Other requirements:** R packages glmnet, data.table

**License:** MIT

**Any restrictions to use by non-academics:** no

## Supplementary information

**Additional file 1.**

## Data Availability

The dataset supporting the conclusions of this article is included within the article and its additional file. Additional file 1 includes the individual signal intensity values, for each dynamic, for each brain region for healthy and diseased dogs. Original image files can be obtained from the main author of this manuscript upon request.

## Abbreviations

AIF: Arterial input function
CBF: Cerebral blood flow
CBV: Cerebral blood volume
CSV: Comma-separated-value
MR: Magnetic resonance
MRI: Magnetic Resonance Imaging
MTT: Mean transit time
PWI: Perfusion weighted imaging
ROI: Region of interest
SVD: Singular value decomposition
T0: Time of arrival
TTP: Time to peak
VOI: volume of interest

## Footnotes

1 R version 3.6.1, R Project for Statistical Computing, Vienna, Austria.

2 Philips Intera Gyroscan, Philips Healthcare, Hamburg, Germany

3 SENSE-flex M coil, Philips Healthcare, Hamburg, Germany

4 OsiriX Light, v.10.0.4, 32-bit; Pixmeo SARL Bernex, Switzerland

5 Horos, v.4.0.0 RC1; The Horos Project, LGPL license at Horosproject.org, sponsored by Nimble Co LLC d/b/a Purview in Annapolis, MD USA
